# Similar outcomes following non-first-degree and first-degree related donor haploidentical hematopoietic cell transplantation for acute leukemia patients in complete remission: a study from the Global Committee and the Acute Leukemia Working Party of the European Society for Blood and Marrow Transplantation

**DOI:** 10.1186/s13045-023-01421-9

**Published:** 2023-03-18

**Authors:** Yishan Ye, Myriam Labopin, Jia Chen, Zafer Gulbas, Xi Zhang, Yener Koc, Didier Blaise, Fabio Ciceri, Emmanuelle Polge, Mohamed Houhou, Lin Li, Yi Luo, Depei Wu, He Huang, Mohamad Mohty, Norbert-Claude Gorin

**Affiliations:** 1 Bone Marrow Transplantation Center, The First Affiliated Hospital, Zhejiang University School of Medicine, 79 Qingchun Road, Hangzhou, China; 2 EBMT Global Committee (Shanghai and Paris Offices) and Acute Leukemia Working Party, Hospital Saint-Antoine APHP and Sorbonne University, Paris, France; 3 National Clinical Research Center for Hematologic Diseases, Jiangsu Institute of Hematology, The First Affiliated Hospital of Soochow University, Suzhou, China; 4 Anadolu Medical Center Hospital Bone Marrow Transplantation Department, Kocaeli, Turkey; 5 Medical Center of Hematology, Xinqiao Hospital, State Key Laboratory of Trauma, Burn and Combined Injury, Army Medical University, Chongqing, China; 6 Medicana International Hospital Istanbul, Bone Marrow Transplant Unit, Istanbul, Turkey; 7 Programme de Transplantation & Therapie Cellulaire, Centre de Recherche en Cancérologie de Marseille, Institut Paoli Calmettes, Marseille, France; 8 Ospedale San Raffaele S.R.L., Haematology and BMT, Milan, Italy; 9 Department of Hematology and Cell Therapy, Hospital Saint-Antoine, Sorbonne University, Paris, France; 10 Service d’Hématologie Clinique et de Thérapie Cellulaire, Hôpital Saint Antoine, APHP, 184 rue du Faubourg Saint-Antoine, 75012 Paris, France

**Keywords:** Haploidentical hematopoietic cell transplantation, Non-first-degree related donor, First-degree related donor

## Abstract

There are situations where non-first-degree (NFD) related donors have to be considered as alternatives to first-degree (FD) related donors for haploidentical hematopoietic cell transplantation (HAPLO). However, the efficacy of these NFD related transplants remains uncertain. All consecutive adult patients (≥ 18 years) with acute myelogenous leukemia (AML) or acute lymphocytic leukemia (ALL) in CR who underwent a first HAPLO between 2010 and 2021 in the European Society for Blood and Marrow Transplantation (EBMT) registry were analyzed. Exact matching and propensity score matching was used. The NFD-to-FD ratio was 1:3. 2703 patients (AML: n = 2047; ALL: n = 656) in CR received a first HAPLO from either NFD (n = 154) or FD (n = 2549) related donors in 177 EBMT centers. 123 NFD and 324 FD HAPLO were included for analysis after matching. Median patient age was 35.6 and 37.2 for the NFD and FD cohorts, respectively. Both cohorts reached good engraftment rates (NFD: 95.7% vs. FD, 95.6%; p = 0.78). The 2-year relapse incidence (NFD, 21.1% vs. FD, 22.6%; p = 0.84) and non-relapse mortality (NRM) (NFD, 13.2% vs. FD, 17.7%; p = 0.33) were not significantly different. The 2-year overall survival (OS) (NFD, 71.8% vs. FD, 68.3%; p = 0.56), leukemia-free survival (LFS) (NFD, 65.7% vs. FD, 59.7%; p = 0.6) and graft-versus-host disease (GVHD)-free, relapse-free survival (GRFS) (NFD, 50.9% vs. FD, 47.8%; p = 0.69) also showed no significant differences. The two cohorts showed no difference in terms of cumulative day 180 grade II-IV, grade III-IV acute GVHD, 2-year cumulative incidences of chronic and extensive chronic GVHD. For HAPLO in patients with acute leukemia, NFD related donors could be equivalent substitutions when FD related donors are not available.

**Supplementary Information:**

The online version contains supplementary material available at 10.1186/s13045-023-01421-9.

**To the editor**,

The choice of donor is a major issue when planning an allogeneic hematopoietic cell transplantation (allo-HCT). The recent development of T-cell-replete haploidentical allo-HCT (HAPLO) pioneered almost simultaneously with different approaches by the Baltimore [1, 2] and the Beijing team [3, 4] has stirred up the field and the proportion of HAPLO among allo-HCTs has surged up globally [5]. The majority of donors for HAPLO are first-degree (FD) related family members such as parents, children or siblings. If a suitable FD related donor is not available, non-first-degree (NFD) related donors, or in very rare occasions related donors with more than 5/10 HLA mismatch [6], have sometimes been used. Previous small studies performed by either the Baltimore [7] or the Beijing team [8, 9] have indicated the feasibility of NFD HAPLO. In a multi-center retrospective study conducted exclusively in China, we observed that NFD and FD HAPLO achieved similar survival transplant outcomes with similar incidences of acute and chronic graft-versus-host disease (aGVHD and cGVHD, respectively) [10]. Other studies however, have reported that NFD HAPLO is associated with slower engraftment and higher incidence of extensive cGVHD [8]. However, all the studies mentioned above have suffered from small sample size and/or with no possible comparative analysis.

The EBMT Global Committee and the Acute Leukemia Working Party (ALWP) of the EBMT therefore decided to compare retrospectively the outcomes of NFD and FD HAPLO, using the EBMT registry in a large cohort of 2703 acute leukemia patients in complete remission (CR), reported by 177 participating centers from January 2010 to January 2021.

Additional file 1: Table S1 describes the demographic and transplant characteristics of these patients (AML: n = 2047; ALL: n = 656). Among them, 154 (5.7%) HAPLO used NFD related donors. Exact matching and propensity score matching were used to control for pre-treatment imbalances in observed variables with an NFD-to-FD ratio of 1:3 (Eligibility criteria, endpoints and statistical analysis are described in Additional file 1: Methods). A total of 123 NFD were matched with 324 FD HAPLO. Additional file 1: Table S2 describes the demographic and transplant characteristics of the two cohorts. The median follow-up durations were 36.8 (range: 28.7–46.4) and 41.4 (35.8–47.5) months for the NFD and FD cohorts, respectively.

NFD related donors consisted of 32 male cousins, 40 female cousins, 10 uncles, 4 aunts, 15 nephews, 4 nieces, and 18 with missing information on kinship. The patient median age was 35.6 (18.8–72.4) and 37.2 (range: 18.1–73.6) for the NFD and FD cohorts, respectively. The main diagnosis in both cohorts was AML (NFD, n = 84, 68.3% vs. FD, n = 233, 71.9%). Status at transplant overall was CR1 in 71.6%, CR2 in 25.7% and CR3 in 2.7% of patients. No statistically significant difference was observed in terms of disease risk index (DRI), hematopoietic cell transplantation comorbidity index (HCT-CI), and Karnofsky performance score. Of note, the proportion of female to male combination was lower in the NFD cohort than in the FD cohort (NFD, 21.1% vs. FD, 30.9%; p = 0.041) and there was a higher percentage of cytomegalovirus seropositivity in patients who underwent NFD than FD HAPLOs (NFD, 66.1% vs. FD, 54.9%, p = 0.038). The majority of patients in both cohorts received myeloablative conditioning (NFD, 69.1% vs. FD, 65.4%) (regimen details summarized in Additional file 1: Table S3). The source of stem cells was peripheral blood (PB) alone in 68% and 46% in the NFD and FD cohorts, respectively. The distribution of the different GVHD prevention regimens was comparable between the two cohorts. Both cohorts achieved good engraftment rates (NFD: 95.7% vs. FD, 95.6%; p = 0.78).

In terms of GVHD, the 180-day cumulative incidences of grade II-IV (NFD, 24% vs. FD, 29.1%; HR = 0.8 (95% CI: 0.52–1.23); p = 0.31) and grade III-IV aGVHD (NFD, 10.7% vs. FD, 9.1%; HR = 1.28 (95% CI: 0.68–2.4); p = 0.44) did not differ. Likewise, there was no significant difference in the 2-year cumulative incidences of cGVHD (NFD, 38.9% vs. FD, 31%; HR = 1.17 (0.81–1.69); p = 0.41) and extensive cGVHD (NFD, 15.3% vs. FD, 9.9%; HR = 1.44 (95% CI: 0.82–2.52); p = 0.2) (Table 1 and Fig. 1).

**Table 1:** Transplant outcomes of patients who underwent NFD or FD HAPLO

	2 years					180 days		2 years	
	Relapse	NRM	LFS	OS	GRFS	Acute GVHD II-IV	Acute GVHD III-IV	Chronic GVHD	Ext. chronic GVHD
FD	22.6% [17.8–27.7]	17.7% [13.5–22.3]	59.7% [53.7–65.3]	68.3% [62.5–73.5]	47.8% [41.8–53.6]	29.1% [24–34.4]	9.1% [6.2–12.7]	31% [25.6–36.6]	9.9% [6.6–13.9]
NFD	21.1% [13.9–29.3]	13.2% [7.7–20.2]	65.7% [55.9–73.9]	71.8% [62.3–79.4]	50.9% [41–60]	24% [16.8–31.9]	10.7% [6–17]	38.9% [29.4–48.3]	15.3% [9.1–22.9]
HR (95% CI)	1.04 (0.69–1.58)	0.77 (0.45–1.31)	0.92 (0.66–1.27)	0.9 (0.63–1.28)	0.95 (0.72–1.25)	0.8 (0.52–1.23)	1.28 (0.68–2.4)	1.17 (0.81–1.69)	1.44 (0.82–2.52)
p value	0.84	0.33	0.6	0.56	0.69	0.31	0.44	0.41	0.2

NRM, non-relapse mortality; LFS, leukemia-free survival; OS, overall survival; GRFS, GVHD-free, relapse-free survival; GVHD, graft-versus-host disease; Ext, extensive; FD, first-degree; NFD, non-first-degree; HR, hazard ratio

**Fig. 1:**
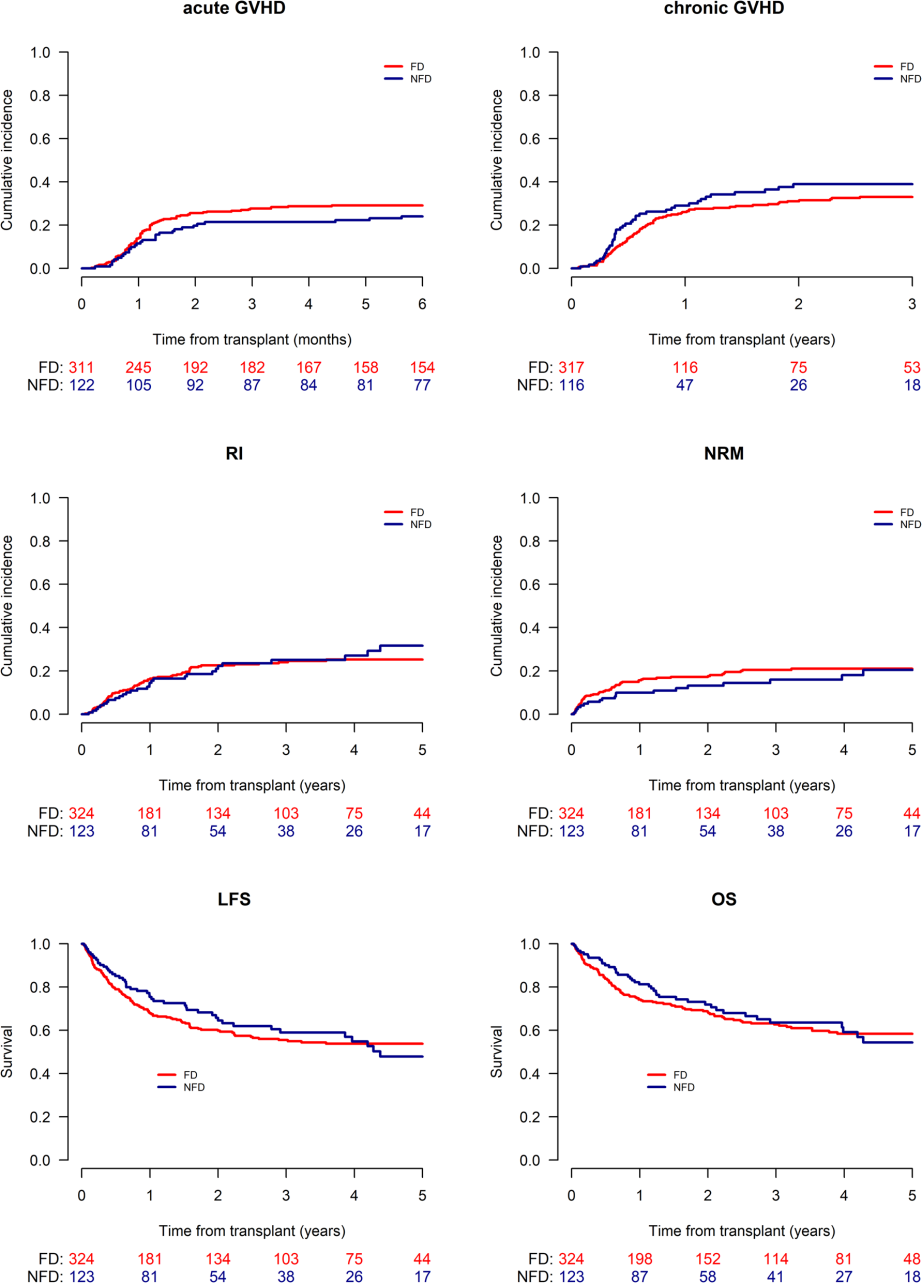
Transplant outcomes comparing the NFD or FD related HAPLOs. The upper part of the Figure shows the 180-day cumulative incidence of grade II-IV acute GVHD and 2-year cumulative incidences of cGVHD comparing the FD (red) and NFD (blue) cohorts. The middle part shows the 2-year cumulative incidence of relapse (RI) and non-relapse mortality (NRM). The lower part shows the 2-year leukemia-free survival (LFS) and overall survival (OS), respectively

There was no difference in the 2-year cumulative incidence of relapse (RI) (NFD, 21.1% vs. FD, 22.6%; HR = 1.04 (95% CI: 0.69–1.58; p = 0.84), NRM (NFD, 13.2% vs. FD, 17.7%; HR = 0.77 (95% CI:0.45–1.31); p = 0.33), LFS (NFD, 65.7% vs. FD, 59.7%; HR = 0.92 (95% CI: 0.66–1.27); p = 0.6) and OS (NFD, 71.8% vs. FD, 68.3%; HR = 0.9 (95% CI: 0.63–1.28; p = 0.56) and in the two cohorts (Table 1 and Fig. 1). Finally, the 2-year GVHD-free, relapse-free survival (GRFS) for both cohorts were similar (NFD, 50.9% vs. FD, 47.8%; HR = 0.95 (95% CI: 0.72–1.25); p = 0.69) (Additional file 1: Figure S1).

A total of 41 patients in the NFD cohort and 110 patients in the FD cohort died during the study period (Additional file 1: Table S4). Original disease was the most common cause of death in both NFD (46.3%) and FD (41.3%) cohorts, followed by infection and GVHD. Other rarer causes of deaths include veno-occlusive disease, engraftment failure, cardiac toxicity, hemorrhage, second malignancy, CNS toxicity and other transplantation related complications.

This analysis which uses pair/propensity score matching, benefits from the largest population of patients so far to receive an NFD HAPLO. Therefore, for HAPLO in patients with acute leukemia, NFD related donors could be equivalent substitutions when FD related donors are not available. We are presently conducting a randomized clinical trial (NCT04547049) to prospectively compare the two donor types for HAPLO.

## Supplementary Information

**Additional file 1. Table S1.** Demographic and transplant characteristics of all patients. **Table S2.** Demographic and transplant characteristics of matched NFD/FD cohorts. **Table S3.** Patients age classes and conditioning regimens. **Table S4.** Cause of death in patients receiving NFD or FD transplants. **Figure S1.** GRFS comparing the NFD and FD related HAPLOs.

## Abbreviations

Allo-HCT: Allogeneic hematopoietic cell transplantation
HAPLO: Haploidentical hematopoietic cell transplantation
FD: First-degree
NFD: Non-first-degree
AML: Acute myeloid leukemia
ALL: Acute lymphocytic leukemia
EBMT: European Society for Blood and Marrow Transplantation
aGVHD: Acute graft-versus-host disease
cGVHD: Chronic graft-versus-host disease
CR: Complete remission
DRI: Disease Risk Index
HCT-CI: Hematopoietic Cell Transplantation Comorbidity Index
RI: Relapse incidence
NRM: Non-relapse mortality
LFS: Leukemia-free survival
OS: Overall survival
GRFS: GVHD-free, relapse-free survival
