# Supplementary material for: Similar outcomes following non-first-degree and first-degree related donor haploidentical hematopoietic cell transplantation for acute leukemia patients in complete remission: a study from the Global Committee and the Acute Leukemia Working Party of the European Society for Blood and Marrow Transplantation
Source: J Hematol Oncol. 2023 Mar 18;16:25. doi: 10.1186/s13045-023-01421-9 (PMC10024403; doi:10.1186/s13045-023-01421-9)
Supplement: Supplementary file 1 — Additional file 1. Table S1. Demographic and transplant characteristics of all patients. Table S2. Demographic and transplant characteristics of matched NFD/FD cohorts. Table S3. Patients age classes and conditioning regimens. Table S4. Cause of death in patients receiving NFD or FD transplants. Figure S1. GRFS comparing the NFD and FD related HAPLOs. [file 13045_2023_1421_MOESM1_ESM.docx]

**Supplementary Appendix**

**Methods**

**Eligibility criteria**

This was a multi-center retrospective analysis using the dataset of the EBMT registry. The EBMT is a voluntary working group of more than 600 transplantation centers. The centers are required to report all consecutive stem cell transplantations and follow-ups once a year. Audits are routinely performed to determine the accuracy of the data. The HCT protocol was approved by the institutional review board of each site, and patients provided informed consent for data collection before transplantation. The study was conducted as per the Declaration of Helsinki and Good Clinical Practice guidelines.

The eligibility criteria for this analysis were as follows: (1) adult patients ≥18 years old; (2) first HAPLO between January 2010 and January 2021; (3) eligible diagnosis: acute myeloid leukemia (AML) and acute lymphoblastic leukemia (ALL) in CR. The exclusion criteria were as follows: allo-HCTs from other donor types (matched sibling donor, matched unrelated donor, mismatched unrelated donor, umbilical cord blood) and previous allo-HCT. The conditioning regimen was defined as myeloablative conditioning (MAC) or reduced-intensity conditioning (RIC) according to the established definitions.[1] GVHD prevention included *ex-vivo* T-cell depletion, anti-T-lymphocyte globulin (ATG), post-transplantation cyclophosphamide (PTCy), or the combination of ATG/PTCy .[2] Myeloid and platelet engraftment was defined as absolute neutrophil count exceeding 0.5×10^9^/L, and platelet count exceeding 20×10^9^/L for 3 consecutive days without transfusion, respectively.

**Endpoints and statistical analysis**

Leukemia-free survival (LFS) was the primary study endpoint. Secondary endpoints were engraftment, overall survival (OS), relapse incidence (RI), non-relapse mortality (NRM), grade II-IV and grade III-IV aGVHD, cGVHD and extensive cGVHD, and GVHD-free, relapse-free survival (GRFS). All endpoints were measured from the date of transplantation.

OS was defined as the time from transplant to death from any cause. LFS was defined as survival with no evidence of relapse or progression. GRFS events were defined as the first event among grade III-IV aGVHD, extensive cGVHD, relapse, and death from any cause. Acute and cGVHD was diagnosed and graded using the revised Glucksberg[3] and the NIH criteria[4], respectively. Patients’ characteristics were compared using the Mann-Whitney test for continuous variables, and the chi-squared or Fisher’s exact test for categorical variables. Exact matching and propensity score (PS) matching were used to control for pre-treatment imbalances in observed variables with an NFD-to-FD ratio of 1:3. Exact matching was performed for diagnosis (ALL or AML), status at transplant (CR1/ CR2/ CR3), age per 5 years, and GVHD prevention (PTCy, ATG, both, *ex-vivo* manipulation). The propensity score was based on patient sex, and conditioning intensity. We checked that patients were well matched with standardized mean difference estimates of less than 5% for all matched parameters. Patients with only 1 or 2 controls were included in the analysis. The probabilities of OS, LFS, and GRFS were calculated using the Kaplan-Meier estimate. The probabilities of RI, NRM, acute and cGVHD were estimated using cumulative incidence curves. Comparisons were performed using a Cox regression or proportional hazards model and **cluster-robust standard errors were used** to account for dependence between observations within matched pairs. Results were expressed as the hazard ratio (HR) with the 95% confidence interval (95% CI). All tests were two-sided with a type 1 error rate fixed at 0.05. Statistical analyses were performed with SPSS 25.0 (IBM Corp., Armonk, NY, USA) and R 4.0.2 (R Core Team (2020. R: A language and environment for statistical computing. R Foundation for Statistical Computing, Vienna, Austria. URL <https://www.R-project.org/>).

**References**

1. Bacigalupo A, Ballen K, Rizzo D, Giralt S, Lazarus H, Ho V, et al. Defining the Intensity of Conditioning Regimens: Working Definitions. Biol Blood Marrow Transplant [Internet]. Elsevier Ltd; 2009;15:1628–33. Available from: http://dx.doi.org/10.1016/j.bbmt.2009.07.004

2. Luo Y, Xiao H, Lai X, Shi J, Tan Y, He J, et al. T-cell-replete haploidentical HSCT with low-dose anti-T-lymphocyte globulin compared with matched sibling HSCT and unrelated HSCT. Blood. 2014;124:2735–43.

3. Harris AC, Young R, Devine S, Hogan WJ, Ayuk F, Bunworasate U, et al. International, Multicenter Standardization of Acute Graft-versus-Host Disease Clinical Data Collection: A Report from the Mount Sinai Acute GVHD International Consortium. Biol Blood Marrow Transplant. 2016;22:4–10.

4. Jagasia MH, Greinix HT, Arora M, Williams KM, Wolff D, Cowen EW, et al. National Institutes of Health Consensus Development Project on Criteria for Clinical Trials in Chronic Graft-versus-Host Disease: I. The 2014 Diagnosis and Staging Working Group Report. Biol Blood Marrow Transplant [Internet]. Elsevier Inc; 2015;21:389-401.e1. Available from: http://dx.doi.org/10.1016/j.bbmt.2014.12.001

**Table S1. Demographic and transplant characteristics of all patients**

|  | **NFD (n=154)** | **FD (n=2549)** |  | P |
| --- | --- | --- | --- | --- |
| **Follow-up months (median,range)** | 29.5 [22.8-37.5] | 38.8 [36.9-41.6] |  | 0.03 |
| **Donor-recipient relation** |  |  |  | < 0.0001 |
| Father |  | 238 |  |  |
| Mother |  | 226 |  |  |
| Brother |  | 600 |  |  |
| Sister |  | 350 |  |  |
| Son |  | 719 |  |  |
| Daughter |  | 390 |  |  |
| Cousin male | 70 |  |  |  |
| Cousin female | 31 |  |  |  |
| Uncle | 11 |  |  |  |
| Aunt | 4 |  |  |  |
| Nephew | 16 |  |  |  |
| Niece | 4 |  |  |  |
| Not specified | 18 | 26 |  |  |
| **Patient age, y (median, range)** | 35.2 (18.8-72.4) | 47.3 (18-75.9) |  | < 0.0001 |
| **Year of transplant**  **(median, range)** | 2018 (2010-2021) | 2017 (2010-2021) |  | < 0.0001 |
| **Diagnosis, n(%)** |  |  |  | < 0.0001 |
| AML | 96 (62.3%) | 1951 (76.5%) |  |  |
| ALL | 58 (37.7%) | 598 (23.5%) |  |  |
| **Status at transplant, n(%)** |  |  |  | 0.44 |
| CR1 | 106 (68.8%) | 1853 (72.7%) |  |  |
| CR2 | 40 (26%) | 605 (23.7%) |  |  |
| CR3 | 8 (5.2%) | 91 (3.6%) |  |  |
| **DRI, n(%)** |  |  |  | 0.11 |
| Low | 12 (7.8%) | 163 (6.4%) |  |  |
| Intermediate | 116 (75.3%) | 1765 (69.4%) |  |  |
| High | 26 (16.9%) | 615 (24.2%) |  |  |
| Missing | 0 | 6 |  |  |
| **HCT-CI, n(%)** |  |  |  | 0.11 |
| HCT-CI = 0 | 55 (70.5%) | 1129 (59.4%) |  |  |
| HCT-CI = 1 or 2 | 9 (11.5%) | 376 (19.8%) |  |  |
| HCT-CI >=3 | 14 (17.9%) | 397 (20.9%) |  |  |
| missing | 76 | 647 |  |  |
| **AML, cytogenetics** |  |  |  | 0.028 |
| Favorable | 11 (14.5%) | 163 (9.4%) |  |  |
| Intermediate | 50 (65.8%) | 1200 (68.9%) |  |  |
| Adverse | 15 (19.7%) | 378 (21.7%) |  |  |
| NA/failed | 20 | 210 |  |  |
| **ALL** |  |  |  | 0.074 |
| Ph negative B-ALL | 12 (26.7%) | 216 (40.3%) |  |  |
| Ph positive B-ALL | 23 (51.1%) | 186 (34.7%) |  |  |
| T-ALL | 10 (22.2%) | 134 (25%) |  |  |
| missing | 45 | 62 |  |  |
| **Karnofsky score, n(%)** |  |  |  | < 0.0001 |
| <90 | 58 (52.7%) | 750 (32%) |  |  |
| >=90 | 52 (47.3%) | 1594 (68%) |  |  |
| missing | 44 | 205 |  |  |
| **Patient sex, n(%)** |  |  |  | 0.65 |
| Male | 95 (61.7%) | 1524 (59.9%) |  |  |
| Female | 59 (38.3%) | 1022 (40.1%) |  |  |
| Missing | 0 | 3 |  |  |
| **Donor sex, n(%)** |  |  |  | 0.081 |
| Donor male | 106 (68.8%) | 1575 (61.8%) |  |  |
| Donor female | 48 (31.2%) | 973 (38.2%) |  |  |
| Missing | 0 | 1 |  |  |
| **Female to male combination, n(%)** |  |  |  | 0.17 |
| No | 126 (81.8%) | 1963 (77.1%) |  |  |
| Yes | 28 (18.2%) | 583 (22.9%) |  |  |
| Missing | 0 | 3 |  |  |
| **Patient CMV, n(%)** |  |  |  | 0.006 |
| Pat. CMV sero-negative | 52 (38.8%) | 688 (27.7%) |  |  |
| Pat. CMV sero-positive | 82 (61.2%) | 1794 (72.3%) |  |  |
| missing | 20 | 67 |  |  |
| **Donor CMV, n(%)** |  |  |  | < 0.0001 |
| Don. CMV sero-negative | 65 (57.5%) | 956 (38.9%) |  |  |
| Don. CMV sero-positive | 48 (42.5%) | 1503 (61.1%) |  |  |
| missing | 41 | 90 |  |  |
| **Conditioning, n(%)** |  |  |  | < 0.0001 |
| MAC | 103 (72%) | 1380 (54.5%) |  |  |
| RIC | 40 (28%) | 1153 (45.5%) |  |  |
| Missing | 11 | 16 |  |  |
| **Detailed conditioning regimen** |  |  |  |  |
| BuCy | 276 (10.9%) | 80 (51.9%) |  |  |
| BuFlu | 413 (16.3%) | 13 (8.4%) |  |  |
| TBF | 878 (34.7%) | 27 (17.5%) |  |  |
| FluMel | 67 (2.6%) | 1 (0.6%) |  |  |
| FLAMSA-Mel | 3 (0.1%) | 0 (0%) |  |  |
| FTM | 72 (2.8%) | 1 (0.6%) |  |  |
| FluTreo | 109 (4.3%) | 4 (2.6%) |  |  |
| FluCy | 14 (0.6%) | 1 (0.6%) |  |  |
| Cy-TBI | 28 (1.1%) | 5 (3.2%) |  |  |
| Flu-TBI | 592 (23.4%) | 16 (10.4%) |  |  |
| Bu-TBI | 29 (1.1%) | 1 (0.6%) |  |  |
| FLAMSA-TBI | 6 (0.2%) | 0 (0%) |  |  |
| TBI-VP16 | 13 (0.5%) | 1 (0.6%) |  |  |
| TBI-other | 5 (0.2%) | 0 (0%) |  |  |
| Bu-Mel | 1 (0%) | 0 (0%) |  |  |
| Thiotepa-based | 2 (0.1%) | 0 (0%) |  |  |
| Other conditioning regimen | 25 (1%) | 4 (2.6%) |  |  |
| Missing | 16 | 0 |  |  |
| **GVHD prevention, n(%)** |  |  |  | < 0.0001 |
| T-cell replete PTCy | 62 (41.3%) | 1639 (70.2%) |  |  |
| T-cell replete ATG | 74 (49.3%) | 408 (17.5%) |  |  |
| T-cell replete PTCy+ATG | 1 (0.7%) | 134 (5.7%) |  |  |
| Ex-vivo T cell depletion | 13 (8.7%) | 154 (6.6%) |  |  |
| Missing | 4 | 214 |  |  |
| **Cell source, n(%)** |  |  |  |  |
| BM | 23 | 703 |  |  |
| PB | 102 | 1593 |  |  |
| BM+PB | 4 | 67 |  |  |
| BM+CB | 3 | 29 |  |  |
| PB+CB | 13 | 37 |  |  |
| BM+PB+CB | 9 | 119 |  |  |
| **Engraftment after HCT, n(%)** |  |  |  | 0.19 |
| graft failure | 4 (2.6%) | 129 (5.4%) |  |  |
| engrafted | 149 (97.4%) | 2260 (94.6%) |  |  |
| missing | 1 | 160 |  |  |
| **Acute GVHD, n(%)** |  |  |  |  |
| Grade I | 29 (19.5%) | 385 (15.5%) |  |  |
| Grade II | 20 (13.4%) | 474 (19%) |  |  |
| Grade III | 9 (6%) | 167 (6.7%) |  |  |
| Grade IV | 8 (5.4%) | 73 (2.9%) |  |  |
| Present, grade unknown | 0 (0%) | 34 (1.4%) |  |  |
| No aGvHD present (Grade 0) | 83 (55.7%) | 1357 (54.5%) |  |  |
| missing | 5 | 59 |  |  |

**Abbreviations:** NFD, non-first-degree; FD, first-degree; DRI, Disease Risk Index; HCT-CI, Hematopoietic Cell Transplantation Comorbidity Index; MAC, myeloablative; RIC, Reduced intensity conditioning; Bu, busulfan; Cy, cyclophosphamide; TBF, thiotepa/busulfan/fludarabine based regimen; Mel, melphalan; FLAMSA, fludarabine/cytarabine/amsacrine based regimen; FTM, fludarabine/carmustin/thiotepa based regimen; FluTreo, fludarabine/treosulfan based regimen; TBI, total body irradiation; VP16, etoposide; PTCy: post-transplant cyclophosphamide; ATG: anti-thymocyte globulin; BM, bone marrow; PB, peripheral blood; CB, cord blood; GVHD, graft-versus-host disease

**Table S2 Demographic and transplant characteristics of matched NFD/FD cohorts**

|  | **NFD (n=123)** | **FD (n=324)** |  | **P** |
| --- | --- | --- | --- | --- |
| **Follow-up: months, median (range)** | 36.8 (28.7-46.4) | 41.4 (35.8-47.5) |  |  |
| **Donor-recipient relationship** |  |  |  |  |
| Father |  | 51 |  |  |
| Mother |  | 45 |  |  |
| Brother |  | 59 |  |  |
| Sister |  | 58 |  |  |
| Son |  | 62 |  |  |
| Daughter |  | 46 |  |  |
| Cousin male | 32 |  |  |  |
| Cousin female | 40 |  |  |  |
| Uncle | 10 |  |  |  |
| Aunt | 4 |  |  |  |
| Nephew | 15 |  |  |  |
| Niece | 4 |  |  |  |
| Not specified | 18 | 3 |  |  |
| **Patient age: y, median (range)** | 35.6 (18.8-72.4) | 37.2 (18.1-73.6) |  | 0.48 |
| **Year of transplant:**  **median (range)** | 2018 (2010-2021) | 2016 (2010-2021) |  | 0.0004 |
| **Diagnosis, n (%)** |  |  |  |  |
| AML | 84 (68.3) | 233 (71.9) |  | 0.45 |
| ALL | 39 (31.7) | 91 (28.1) |  |  |
| **Status at transplant, n (%)** |  |  |  |  |
| CR1 | 86 (69.9) | 234 (72.2) |  | 0.8 |
| CR2 | 33 (26.8) | 82 (25.3) |  |  |
| CR3 | 4 (3.3) | 8 (2.5) |  |  |
| **DRI, n (%)** |  |  |  |  |
| Low | 11 (8.9) | 17 (5.3) |  | 0.34 |
| Intermediate | 93 (75.6) | 249 (77.3) |  |  |
| High | 19 (15.4) | 56 (17.4) |  |  |
| Missing | 0 | 2 |  |  |
| **HCT-CI, n (%)** |  |  |  |  |
| HCT-CI = 0 | 44 (67.7) | 118 (54.1) |  | 0.11 |
| HCT-CI = 1 or 2 | 7 (10.8) | 44 (20.2) |  |  |
| HCT-CI >=3 | 14 (21.5) | 56 (25.7) |  |  |
| Missing | 58 | 106 |  |  |
| **Karnofsky score, n (%)** |  |  |  |  |
| <90 | 47 (49) | 125 (44.8) |  | 0.48 |
| >=90 | 49 (51) | 154 (55.2) |  |  |
| Missing | 27 | 45 |  |  |
| **Patient sex, n (%)** |  |  |  |  |
| Male | 82 (66.7) | 211 (65.1) |  | 0.76 |
| Female | 41 (33.3) | 113 (34.9) |  |  |
| **Donor sex, n (%)** |  |  |  |  |
| Male | 83 (67.5) | 175 (54) |  | 0.01 |
| Female | 40 (32.5) | 149 (46) |  |  |
| **Female to male combination, n (%)** |  |  |  |  |
| No | 97 (78.9) | 224 (69.1) |  | 0.041 |
| Yes | 26 (21.1) | 100 (30.9) |  |  |
| **Patient CMV, n (%)** |  |  |  |  |
| Patient CMV sero-negative | 39 (33.9) | 142 (45.1) |  | 0.038 |
| Patient CMV sero-positive | 76 (66.1) | 173 (54.9) |  |  |
| Missing | 8 | 9 |  |  |
| **Donor CMV, n (%)** |  |  |  |  |
| Donor CMV sero-negative | 49 (50.5) | 156 (50.3) |  | 0.97 |
| Donor CMV sero-positive | 48 (49.5) | 154 (49.7) |  |  |
| Missing | 26 | 14 |  |  |
| **Conditioning, n (%)** |  |  |  |  |
| MAC | 85 (69.1) | 212 (65.4) |  | 0.46 |
| RIC | 38 (30.9) | 112 (34.6) |  |  |
| **GVHD prevention, n (%)** |  |  |  |  |
| T-cell replete PTCy | 61 (49.6) | 181 (55.9) |  | 0.48 |
| T-cell replete ATG | 49 (39.8) | 114 (35.2) |  |  |
| T-cell replete PTCy+ATG | 1 (0.8) | 1 (0.3) |  |  |
| Ex-vivo T cell depletion | 12 (9.8) | 28 (8.6) |  |  |
| **Cell source, n (%)** |  |  |  |  |
| BM | 20 (16.3) | 78 (24.1) |  |  |
| PB | 84 (68.3) | 150 (46.3) |  |  |
| BM+PB | 4 (3.3) | 16 (4.9) |  |  |
| BM+CB | 3 (2.4) | 12 (3.7) |  |  |
| PB+CB | 6 (4.9) | 14 (4.3) |  |  |
| BM+PB+CB | 6 (4.9) | 54 (16.7) |  |  |
| **Engraftment after HCT, n (%)** |  |  |  |  |
| Graft failure | 4 (3.3) | 12 (4.4) |  | 0.78 |
| Engrafted | 118 (96.7) | 258 (95.6) |  |  |
| Missing | 1 | 54 |  |  |
| **Acute GVHD, n (%)** |  |  |  |  |
| Grade I | 27 (22.1) | 48 (15.3) |  |  |
| Grade II | 16 (13.1) | 59 (18.8) |  |  |
| Grade III | 6 (4.9) | 20 (6.4) |  |  |
| Grade IV | 8 (6.6) | 7 (2.2) |  |  |
| Grade unknown | 0 (0) | 3 (1) |  |  |
| No aGVHD present (Grade 0) | 65 (53.3) | 177 (56.4) |  |  |
| Missing | 1 | 10 |  |  |

**Abbreviations:** NFD, non-first-degree; FD, first-degree; DRI, Disease Risk Index; HCT-CI, Hematopoietic Cell Transplantation Comorbidity Index; MAC. Myeloablative conditioning; RIC: Reduced intensity conditioning; PTCy: post-transplant cyclophosphamide; ATG: anti-thymocyte globulin; BM, bone marrow; PB, peripheral blood; CB, cord blood; GVHD, graft-versus-host disease; CMV, cytomegalovirus; CR, complete remission

**Table S3: Patients age classes and conditioning regimens**

| **Details** | **NFD (n=123)** | **FD (n=324)** |
| --- | --- | --- |
| Age classes, n(%) |  |  |
| 18-24 | 21 (17.1%) | 60 (18.5%) |
| 25-29 | 16 (13%) | 36 (11.1%) |
| 30-34 | 18 (14.6%) | 42 (13%) |
| 35-39 | 22 (17.9%) | 52 (16%) |
| 40-44 | 14 (11.4%) | 40 (12.3%) |
| 45-49 | 4 (3.3%) | 12 (3.7%) |
| 50-54 | 9 (7.3%) | 27 (8.3%) |
| 55-59 | 4 (3.3%) | 12 (3.7%) |
| 60-64 | 6 (4.9%) | 18 (5.6%) |
| 65+ | 9 (7.3%) | 25 (7.7%) |
| **Details of conditioning regimens, n(%)** | | |
| BuCy | 54 (43.9%) | 99 (30.6%) |
| BuFlu | 13 (10.6%) | 19 (5.9%) |
| TBF | 26 (21.1%) | 100 (30.9%) |
| FluMel | 1 (0.8%) | 5 (1.5%) |
| FLAMSA-Mel | 0 (0%) | 1 (0.3%) |
| FTM | 1 (0.8%) | 11 (3.4%) |
| FluTreo | 4 (3.3%) | 12 (3.7%) |
| FluCy | 1 (0.8%) | 0 (0%) |
| Cy-TBI | 4 (3.3%) | 2 (0.6%) |
| Flu-TBI | 14 (11.4%) | 64 (19.8%) |
| Bu-TBI | 1 (0.8%) | 7 (2.2%) |
| TBI-VP16 | 1 (0.8%) | 2 (0.6%) |
| Other conditioning regimen | 3 (2.4%) | 2 (0.6%) |

**Abbreviations:** Bu, Busulfan; Cy, cyclophosphamide; Flu, fludarabine; TBF, thiotepa/busulfan/fludarabine based regimen; Mel, melphalan; FLAMSA, fludarabine/cytarabine/amsacrine based regimen; FTM, fludarabine/carmustin/thiotepa based regimen; FluTreo, fludarabine/treosulfan based regimen; TBI, total body irradiation; VP16, etoposide;

**Table S4. Cause of death in patients receiving NFD or FD transplants**

|  | **NFD (n=41)** | **FD (n=110)** |
| --- | --- | --- |
| Cause n (%)  Original disease | 19 (46.3) | 43 (41.3) |
| GVHD | 12 (29.3) | 13 (12.5) |
| Infection | 6 (14.6) | 31 (29.8) |
| Non HCT related | 4 (9.8) | 3 (2.9) |
| Cardiac toxicity | 0 (0) | 1 (1) |
| Hemorrhage | 0 (0) | 1 (1) |
| Failure/Rejection | 0 (0) | 2 (1.9) |
| VOD | 0 (0) | 4 (3.8) |
| Other second malignancy | 0 (0) | 1 (1) |
| CNS toxicity | 0 (0) | 1 (1) |
| Other transplant related | 0 (0) | 4 (3.8) |
| Missing | 0 | 6 |

**Abbreviations:** GVHD, graft-versus-host disease; HCT, hematopoietic cell transplantation; VOD, veno-occlusive disease; CNS, central nervous system; NFD, non-first-degree; FD, first-degree

**Figure S1. GRFS comparing the NFD and FD related HAPLOs**

**
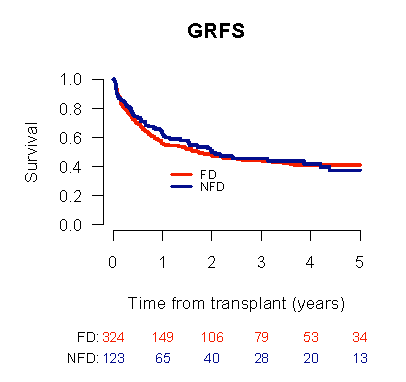
**

Figure S1. The 2-year GVHD-free, relapse-free survival (GRFS) for comparing the FD (red) and NFD (blue) cohorts.
